# A Novel Tool for Real-time Estimation of Epidemiological Parameters of Communicable Diseases Using Contact-Tracing Data: Development and Deployment

**DOI:** 10.2196/34438

**Published:** 2022-05-31

**Authors:** Bernard C Silenou, Carolin Verset, Basil B Kaburi, Olivier Leuci, Stéphane Ghozzi, Cédric Duboudin, Gérard Krause

**Affiliations:** 1 Department of Epidemiology Helmholtz Centre for Infection Research Braunschweig Germany; 2 Hannover Medical School Hannover Germany; 3 Agence Régionale de Santé de Bourgogne Franche-Comté Dijon France; 4 German Center for Infection Research Braunschweig Germany

**Keywords:** COVID-19, disease outbreak, contact tracing, serial interval, basic reproduction number, infectious disease incubation period, superspreading events, telemedicine, public health, epidemiology, surveillance tool, outbreak response, pandemic, digital health application, response strategy

## Abstract

**Background:**

The Surveillance Outbreak Response Management and Analysis System (SORMAS) contains a management module to support countries in their epidemic response. It consists of the documentation, linkage, and follow-up of cases, contacts, and events. To allow SORMAS users to visualize data, compute essential surveillance indicators, and estimate epidemiological parameters from such network data in real-time, we developed the SORMAS Statistics (SORMAS-Stats) application.

**Objective:**

This study aims to describe the essential visualizations, surveillance indicators, and epidemiological parameters implemented in the SORMAS-Stats application and illustrate the application of SORMAS-Stats in response to the COVID-19 outbreak.

**Methods:**

Based on findings from a rapid review and SORMAS user requests, we included the following visualization and estimation of parameters in SORMAS-Stats: transmission network diagram, serial interval (SI), time-varying reproduction number R(t), dispersion parameter k, and additional surveillance indicators presented in graphs and tables. We estimated SI by fitting lognormal, gamma, and Weibull distributions to the observed distribution of the number of days between symptom onset dates of infector-infectee pairs. We estimated k by fitting a negative binomial distribution to the observed number of infectees per infector. Furthermore, we applied the Markov Chain Monte Carlo approach and estimated R(t) using the incidence data and the observed SI computed from the transmission network data.

**Results:**

Using COVID-19 contact-tracing data of confirmed cases reported between July 31 and October 29, 2021, in the Bourgogne-Franche-Comté region of France, we constructed a network diagram containing 63,570 nodes. The network comprises 1.75% (1115/63,570) events, 19.59% (12,452/63,570) case persons, and 78.66% (50,003/63,570) exposed persons, including 1238 infector-infectee pairs and 3860 transmission chains with 24.69% (953/3860) having events as the index infector. The distribution with the best fit to the observed SI data was a lognormal distribution with a mean of 4.30 (95% CI 4.09-4.51) days. We estimated a dispersion parameter k of 21.11 (95% CI 7.57-34.66) and an effective reproduction number R of 0.9 (95% CI 0.58-0.60). The weekly estimated R(t) values ranged from 0.80 to 1.61.

**Conclusions:**

We provide an application for real-time estimation of epidemiological parameters, which is essential for informing outbreak response strategies. The estimates are commensurate with findings from previous studies. The SORMAS-Stats application could greatly assist public health authorities in the regions using SORMAS or similar tools by providing extensive visualizations and computation of surveillance indicators.

## Introduction

### Background

During the course of 2020, there was a substantial increase in the number and use of eHealth applications, mainly in response to the COVID-19 outbreak [1-3]. These applications are being used in different areas of digital health intervention, such as disease surveillance, vaccine delivery, hospital management, laboratory management, symptom journals, and education [4].

The Surveillance Outbreak Response Management and Analysis System (SORMAS) is an open-source digital tool that supports disease control and outbreak management procedures [5-8]. The objective of SORMAS is to ensure the availability of real-time surveillance data for priority diseases at all administrative levels. SORMAS supports task management, complies with data protection and data security standards, and enhances interoperability with other applications.

Essential epidemiological parameters governing COVID-19 transmissions such as serial interval (SI), instantaneous reproduction number R(t), effective reproduction number R, and individual-level variation in transmission are context-specific, and thus often difficult to estimate precisely with publicly available data. Studies have been conducted to estimate these epidemiological parameters during the early phase of the outbreak, but most focused on coarse, aggregated, and publicly reported data sources that likely mask local specificities or are biased toward more severe cases [9-11]. The lack of easy access to outbreak data containing more epidemiological and clinical information has been reported as a limiting factor in improving the performance of epidemiological models [12]. Since then, most public health stakeholders used eHealth applications to document rich and large contact-tracing data in response to the COVID-19 outbreak. Nonetheless, such informative surveillance data are often not hosted on a centralized hub but scattered on different databases in the corresponding countries. In addition, the estimation of epidemiological parameters is frequently not done in real time and does not account for spatial and temporal variation, and thus, fails to provide comprehensive and timely information on outbreak evolution to best inform decision-making [13]. Further, public health stakeholders faced challenges in generating reproducible analyses for their routine situation reports because data are often manually exported from eHealth applications and analyzed with stand-alone statistical applications such as R, SAS, SPSS or STATA.

To address these challenges, we envisaged an approach whereby standardized statistical analysis methods are brought to where the rich and detailed surveillance data are hosted. To assist stakeholders (SORMAS users) with a reactive analytic platform that leverages the rich and detailed data documented in SORMAS, we developed the SORMAS Statistics (SORMAS-Stats) application. SORMAS-Stats is a user-friendly R Shiny web application to estimate epidemiological parameters, compute country or region-specific indicators, and provide visualizations in real time.

### Objective

This study aims to describe the essential visualizations, epidemiological parameters, and surveillance indicators implemented in the SORMAS-Stats application. We illustrated its application in response to the COVID-19 outbreak using surveillance data captured with SORMAS in the Bourgogne-Franche-Comté region of France.

## Methods

### Design Process of SORMAS-Stats

We gathered the requirements essential to supporting outbreak response through SORMAS user requests (from Nigeria, Ghana, France, and Germany), which thus guided the design of SORMAS-Stats. SORMAS users are public health personnel of a country, such as field investigators and epidemiologists. We identified user requests through GitHub issues created by SORMAS users, the SORMAS user support platform, and the minutes of sprint planning meetings. The sprint planning meetings took place every 3 weeks, matching the software release cycle of SORMAS. In parallel, we conducted a rapid review of epidemiological publications and situation reports. Subsequently, we combined the essential requirements obtained from the review and the users’ requests, implemented them, and released a beta version of the SORMAS-Stats application. Further, we conducted a field test of the beta version, got users’ feedback, implemented them, and deployed a stable version. The time from requirement gathering to deployment was 12 months (July 2020 to June 2021).

### Overview of SORMAS-Stats

SORMAS-Stats is a web application that can be installed locally and uses advanced visualization and statistical analysis methods to analyze surveillance data in real time. SORMAS-Stats assists public health officials in managing outbreaks and permits the execution of reproducible routine epidemiological analysis. The workflow of SORMAS-Stats consists of the preprocessing phase and the analytics phase.

In the preprocessing phase, SORMAS-Stats imports pseudonymized data from an external database. The default integration of SORMAS-Stats is with the SORMAS PostgreSQL database. Only the records and associated attributes reported within the time interval specified in the SORMAS-Stats configuration file are extracted from the external database. Further data processing steps are the deletion of error records, deduplication, categorization, and the computation of derived variables. In the analytics phase, SORMAS-Stats analyzes the preprocessed data. We classified the analytics phase into two types: (1) data visualization and the computation of summary statistics and (2) the estimation of essential disease-specific epidemiological parameters through statistical modeling.

### Epidemiological Data

The SORMAS-Stats application analyzes the entity-based surveillance data routinely collected by public health workers. Generally, surveillance data include the following entities: case person (a person infected with a disease), contact person (a noninfected person exposed to a case person), and event (any exposure or gathering that poses a threat to human health or may lead to the spread of diseases). The collection of all probable transmission chains (pairs of entities: case person–contact person or event–persons exposed to an event [event participants], that can result in disease transmission) forms the network data of the disease. During contact follow-up, a contact person or event participant may become symptomatic and meet the case definition of the disease in question, thus being converted or reclassified as a case. In such instances, 2 types of infector (ie, index case) and infectee (ie, secondary case) pairs are formed (infector-infectee pairs): first, between the case person and contact person, and second, between the event and the event participant.

The data used to illustrate the application of SORMAS-Stats consisted of confirmed cases of COVID-19 and their contacts documented using SORMAS between July 31 and October 29, 2021, in the Bourgogne-Franche-Comté region of France.

### Estimation of Epidemiological Parameters and Surveillance Indicators

#### Serial Interval

We computed the observed SI as the difference in the number of days between symptom onsets of infector-infectee pairs. We excluded infector-infectee pairs for which the infector was an event or if one of a pair had missing data for symptom onset date. However, we only included symptomatic transmissions with available data for the date of symptom onset for both infector and infectee in a pair, as transmission data are often generated during contact-tracing under symptomatic settings [14]. We estimated the SI distribution by fitting lognormal, gamma, Weibull, and normal distributions to the observed SI data. The choice of these distributions was based on previous studies [15,16]. For all 4 types of distributions, we excluded observed SI greater than 30 days. For lognormal, gamma, and Weibull distributions, which do not take negative values, we dropped negative values before fitting the distributions. For each fitted distribution, we computed the goodness-of-fit criteria (Akaike information criterion and Bayesian information criterion) and goodness-of-fit statistics (Kolmogorov-Smirnov, Cramer-von Mises, and Anderson-Darling) [17]. We chose the distribution with the best fit by several approaches: the smallest Akaike information criterion, a plot of the density function of the fitted distribution with the histogram of observed data, and a plot of the empirical and theoretical cumulative distribution functions of each fitted model. We calculated the 95% CI of the mean SI using the formula μ ± 1.96 × (σ / √n), where μ is the estimated sample mean, σ the estimated sample SD, and n the sample size. The analysis was performed using the R statistical software (R Foundation for Statistical Computing) package fitdistrplus [17].

#### Instantaneous Reproduction Number

We estimated R(t)—on a weekly basis—using the approach proposed by Cori et al [18,19]. This method mainly requires the incidence and contact-tracing data, which are the types of data captured by SORMAS. We implemented 2 approaches to specify the SI distribution used to estimate R(t). First, a parametric distribution for SI with values for the mean and SD, and second, a parametric distribution for SI and the observed data of infector-infectee pairs. For the second approach, we estimated the SI distribution by applying the Markov Chain Monte Carlo method. The possible choices of the parametric distribution for SI were gamma, Weibull, and lognormal. We computed the summary statistics of the posterior mean and plotted the posterior mean and 95% credible interval for R(t). The analysis was performed using the R statistical software package EpiEstim [19].

#### Variation in Transmission Heterogeneity and Effective Reproduction Number

Using the data for infector-infectee pairs of cases, we computed the observed offspring distribution as the number of infectees per infector. We applied the approach described by Lloyd-Smith et al [20] and fitted a negative binomial distribution to the observed offspring distribution. We estimated the effective reproduction number R and the variation in transmission heterogeneity as the mean and dispersion parameter k of the negative binomial distribution, respectively [20]. In addition, we computed the median and 95% percentile CI of both parameters using bootstrap. We performed the analysis using the R statistical software package fitdistrplus [17].

#### Visualizations and Surveillance Indicators

We computed 6 surveillance indicators that may be informative in managing disease outbreaks using transmission network data. Table 1 presents the definition of visualizations and surveillance indicators implemented in the SORMAS-Stats application.

**Table 1:** Description and application of epidemiological parameters, surveillance indicators, and visualizations in SORMAS-Stats application.

Name of output or indicator		Description	Applications in disease surveillance
**Epidemiological parameters**			
	Serial interval (SI)	The difference in the number of days between symptom onsets of infector-infectee pairs (see Methods).	To distinguish disease variants, design follow-up and quarantine duration, and determine time window for effective intervention strategies.
	Instantaneous reproduction number R(t)	The average number of infectees per infector at a particular time t (see Methods).	To assess the impact of intervention measures. R(t)>1 signify an increase in infectiousness at time t, R(t)<1 signify a decrease in infectiousness [18].
	Effective reproduction number (R)	The average number of infectees per infector (see Methods).	Similar to R(t).
	Dispersion parameter (k)	A measure of how the number of infectees per infector (offspring distribution) is distributed around the mean value (see Methods).	To assess the evidence of superspreading events or formation of clusters. This can help to devise relevant control measures. Smaller values of k indicate higher levels of dispersion, thus suggesting evidence of superspreading.
**Surveillance indicators**			
	Proportion of exposed persons who became cases	The proportion of exposed persons—among all exposed persons—that converted to cases by exposure types.	To devise relevant control measures similar to the dispersion parameter k. To assess the quality of contact tracing and better allocate resources.
	Proportion of index infectors	The proportion of index infectors among all infector nodes (infector person, infectee person, or event).	To determine the quality of contact-tracing. A smaller proportion indicates a greater coverage of identified linkages between infectors and infectees.
	Variance-to-mean ratio (VMR)	The variance divided by the mean of the observed offspring distribution [21].	Similar to k. VMR>1 indicates higher levels of dispersion and thus signaling evidence of superspreading.
	Edge density	The ratio of the number of edges (links between 2 nodes) and the maximum number of possible edges [22]. It represents how connected the nodes of the network diagram are to each other.	To assess the impact of control measures on overall societal behavior. Higher values may signify higher social interactions.
	Number of individual transmission chains	The total number of transmission chains or index infector nodes in the network diagram.	Similar to proportion of index infectors.
**Visualizations**			
	Network diagram	A directed graph of all disease transmission chains consisting of the following types of nodes: case persons, contact persons, events, and event participants.	To prioritize investigation and follow-up of events with known confirmed cases.
	Time series plots	A bar graph or line plot of entity counts over time (day, week, or month).	To gauge the efficacy of control measures in place and the need to implement new ones.
	Tables	Tables of entity count, proportions, or incidence proportions by administrative area (regions, districts, community).	To target intervention measures to specific areas of the country such as hotspots.
	Charts	Pie charts and bar graphs of entity counts or proportions by entity attributes (eg, age and sex).	To protect vulnerable groups.
	Maps	Spatial-temporal display of entity counts, proportion, and incidence proportion on a map by administrative area (regions, districts, community).	Similar to tables.

### Architecture of SORMAS and SORMAS-Stats

**Figure 1:**
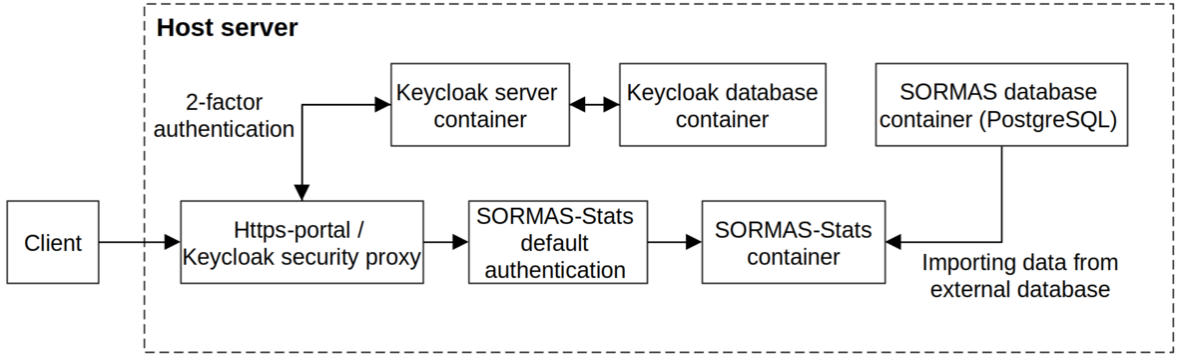
Server setup for the SORMAS-Stats application. SORMAS: Surveillance Outbreak Response Management and Analysis System.

The SORMAS application was developed on the VAADIN framework, JAVA EE, Payara server, and PostgreSQL database. SORMAS consists of 2 components: the mobile app and the web application. The mobile app communicates with the server via a REST-API and the VAADIN web client application. The SORMAS-Stats application analyzes the surveillance data documented in the PostgreSQL database of SORMAS. We developed SORMAS-Stats based on the R Shiny framework [23]. To secure the server hosting the application, we used an architecture with the following configuration: https-portal or secure proxy, 2-factor authentication with Keycloak, and the default authentication of SORMAS-Stats (Figure 1). The default authentication of SORMAS-Stats used the shinyauthr R package authentication module to hash the user password [24,25]. SORMAS-Stats can be executed as a default R Shiny application or a Docker application. We deployed SORMAS, SORMAS PostgreSQL, and SORMAS-Stats applications as separate Docker containers and managed them with one Docker-compose file. The test version of SORMAS-Stats based on demonstration data is available online [26]. The code and description of deployment are hosted on GitHub [27].

### Ethical Considerations

The Agence Régionale de Santé de Bourgogne Franche-Comté, France, as an administrative public health institution, granted permission for using the anonymized COVID-19 outbreak data in this study, under article 11 of Law No. 2020-546 of May 11, 2020. Under this law, the secondary data used in this study did not require individual informed consent.

## Results

### Contents of the SORMAS-Stats User Interface

The SORMAS-Stats application has multiple dashboards with filters, permitting users to execute analysis and download the output. Table 1 depicts the contents of the SORMAS-Stats user interface and their applications in disease surveillance or outbreak management. The contents of the interface are visualizations, epidemiological parameters, and surveillance indicators.

### Description of the Epidemiological Data Used to Illustrate SORMAS-Stats

**Figure 2:**
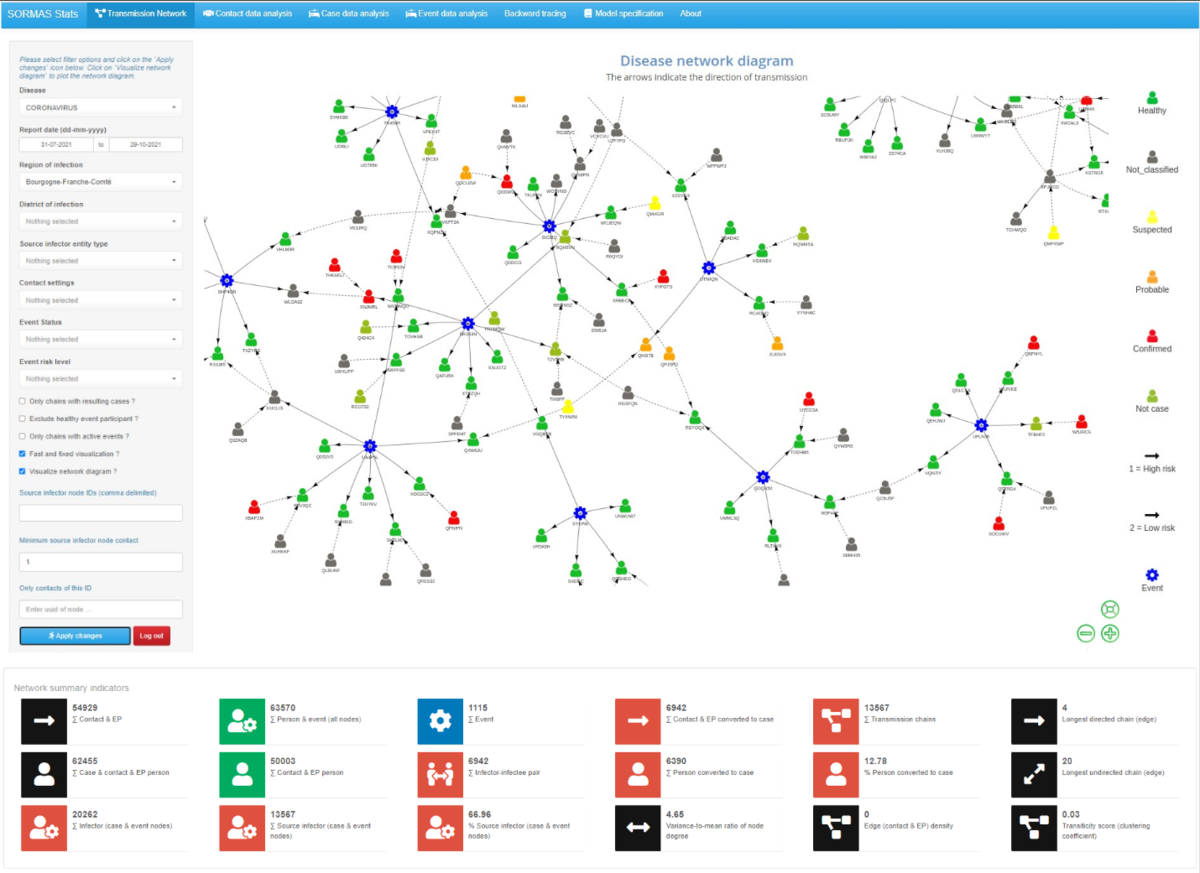
Screenshot of SORMAS-Stats showing the COVID-19 transmission network diagram and surveillance indicators for 63,570 entities reported between July 31 and October 29, 2021, in the Bourgogne-Franche-Comté region of France. The diagram comprises 1115 events (blue gear node), 12,452 case persons (nongreen person node), 50,003 exposed persons (green person node), and 54,929 exposures (directed arrow from infector node). SORMAS-Stats: Surveillance Outbreak Response Management and Analysis System Statistical application.

We used SORMAS-Stats to estimate epidemiological parameters and surveillance indicators by analyzing the contact-tracing data documented in SORMAS between July 31 and October 29, 2021, in the Bourgogne-Franche-Comté region of France. Figure 2 presents a network diagram constructed from the contact-tracing data consisting of 63,570 unique nodes, comprising 1.75% (n=1115) events, 19.59% (n=12,452) case persons, and 78.66% (n=50,003) exposed persons. Of the 50,003 exposed persons, 6390 (12.78%) subsequently converted to cases. The network diagram consisted of 3860 transmission chains, with each chain comprising a minimum of 1 exposed person and a source infector node (case person or event). The length of the longest directed chain was 4 generations of infection. The average number of exposed persons per node (node degree) was 1.73 (IQR 1-228), whereas the variance-to-mean ratio was 4.65.

### Epidemiological Parameters and Surveillance Indicators

After subsetting the network diagram by considering chains with resulting cases only, 10,250 unique nodes remained, comprising 9.30% (n=953) events and 90.70% (n=9297) case persons. Of the 9297 case persons, 68.73% (n=6390) were infectees. There were 3860 transmission chains, with 24.69% (n=953) having an event as the index infector. The average node degree was 1.36 (IQR 1-36), whereas the variance-to-mean ratio was 1.05. The edge density of the complete or reduced network diagram considering chains with only resulting cases was <0.01.

**Figure 3:**
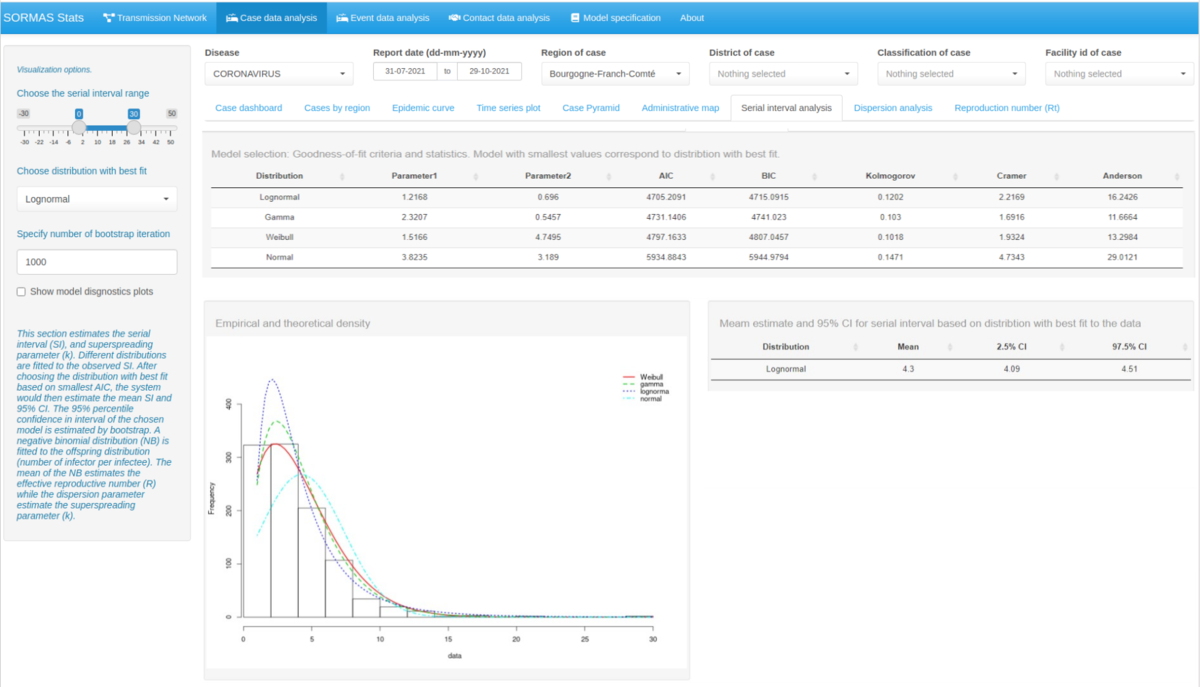
Screenshot of SORMAS-Stats showing the COVID-19 serial interval distribution for 1238 infector-infectee pairs reported between July 31 and October 29, 2021, in the Bourgogne-Franche-Comté region of France. AIC: Akaike information criterion; BIC: Bayesian information criterion; SORMAS-Stats: Surveillance Outbreak Response Management and Analysis System Statistical application.

**Figure 4:**
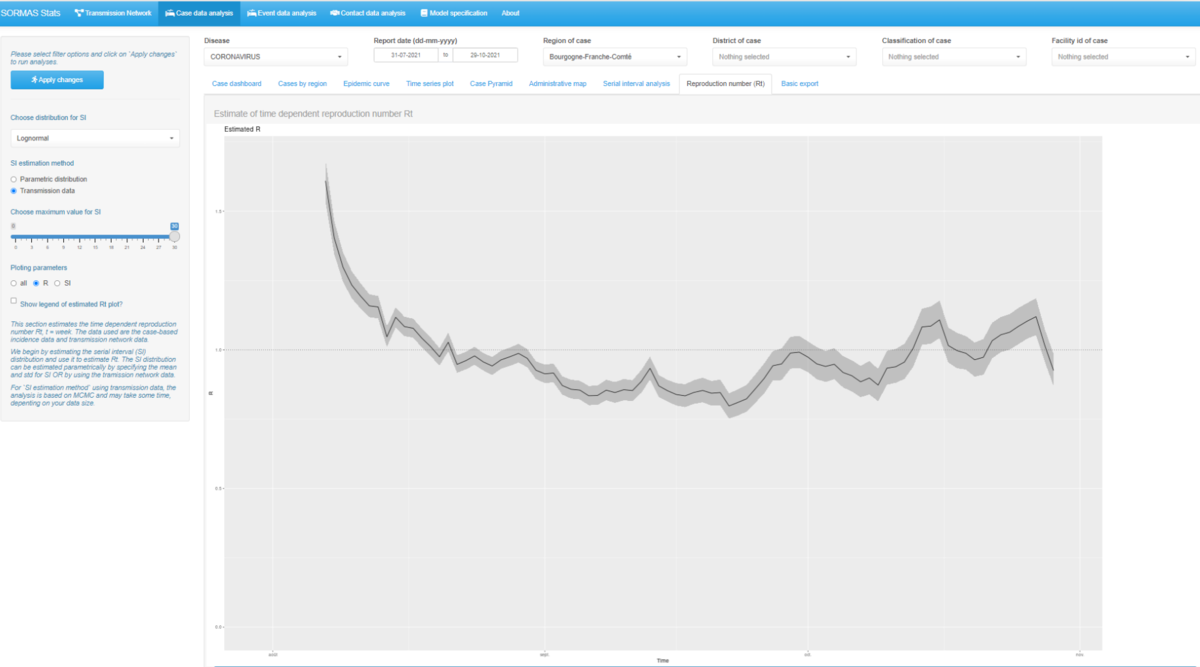
Screenshot of SORMAS-Stats showing an estimate of COVID-19 time-dependent reproduction number (line) with 95% credible interval (grey band) for 12,452 case persons reported between July 31 and October 29, 2021, in the Bourgogne-Franche-Comté region of France. SORMAS-Stats: Surveillance Outbreak Response Management and Analysis System Statistical application.

Considering infector-infectee pairs consisting of person entities only (event nodes excluded) resulted in 1238 infector-infectee pairs, of which 31.26% (n=387) had available data for symptom onset date. Of the 387 pairs with available data for symptom onset date, 20.41% (n=79) were pairs of asymptomatic transmission for which the onset date of the infectee preceded or was on the same date as that of the infector. After excluding negative SIs, the mean of the observed SI was 3.96 (IQR 0-27**)** days. The distribution with the best fit to the observed SI data was the lognormal distribution with a mean of 4.30 (95% CI 4.09-4.51) days (Figure 3). The mean of the observed offspring distribution was 1.36 (IQR 1-36). By fitting a negative binomial distribution to the observed offspring distribution, we estimated a dispersion parameter k of 21.11 (95% CI 7.57-34.66) and a reproduction number R of 0.9 (95% CI 0.58-0.60). Using the observed transmission data with a lognormal distribution for SI, the estimate of the average weekly posterior mean for R(t) was 0.98 (IQR 0.80-1.61) (Figure 4). The estimated range of R(t) was congruent with the values obtained when plugging in values for SI mean and SD obtained from the literature (5.19 and 4.23 days, respectively) [9].

## Discussion

### Principal Findings

We developed and deployed SORMAS-Stats, an open-source web application for real-time visualization and the estimation of epidemiological parameters using contact-tracing data captured with the SORMAS eHealth application. SORMAS-Stats is easy to deploy and requires no programming skills to perform analyses. Some epidemiological parameters included in SORMAS-Stats are SI, time-varying reproduction number R(t), effective reproduction number R, and dispersion parameter k. We illustrated the use of SORMAS-Stats by analyzing the contact-tracing data for COVID-19 captured between July 31 and October 29, 2021, in the Bourgogne-Franche-Comté region of France. The estimated mean SI was 4.30 days; this was commensurate with findings from previous studies [9,11]. The estimate for k was 21.11, whereas the variance-to-mean ratio of the offspring distribution was >1 (1.05), signifying a probable clustering of infections compatible with the observation of superspreading events. However, the estimate was not of the same magnitude as findings from 2 previous studies that used contact-tracing data from other countries at the early phase of the pandemic and reported k of 0.58 and 0.43 [28,29]. This difference in estimates could be related to various factors that may have prevented superspreading events, such as (1) intervention measures (such as the closure of bars, schools, and gatherings) that stakeholders may have enforced during the study period or (2) differences in the geographical area. The possibility of estimating k in real time may assist stakeholders in understanding the current local infection dynamics and thus inform which, if any, control measure would be most appropriate. The weekly estimated R(t) values ranged from 0.80 to 1.61. The maximum value of 1.61 was at the start of the study period; this value was not well estimated since there were no data for the preceding weeks. However, the subsequent values were well estimated and predominantly fluctuated slightly below and above 1, signifying that the infection dynamics were stable within the study period. The computed density of the transmission network diagram was small (<0.01), suggesting low social interactions among the persons in the population, thus resulting in a low transmission rate.

The visualizations included in SORMAS-Stats are maps, charts, tables, time series plots, and network diagrams. Further, SORMAS-Stats contains several filters to explore the network diagram by clusters, transmission chains, and superspreading events. The possibility to filter the network diagram was helpful to stakeholders, not only to assist the exploration of transmission chains but to also detect and correct errors created during the data collection phase. SORMAS-Stats is a stand-alone application, easily deployable owing to Docker technology and does not depend on the principal application or method used for data collection. Thus, users with programming skills in R statistical software only can easily configure and extend SORMAS-Stats to cover other types of statistical analyses.

SORMAS-Stats has a public GitHub repository that permits stakeholders from interested countries to make additional requests and contributions. In this way, it stays an application developed by and for public health workers.

### Limitations

The current integration of SORMAS-Stats is with the SORMAS PostgreSQL database. However, with minor adjustment, SORMAS-Stats can be integrated with other databases or files that can be read by R statistical software as long as the relationship between the entities (such as case person, contact person, event, and event participant) are referenced across the tables in the database [30]. The range of the length of the directed transmission chain was 1 to 4; the majority of the chains had 2 or fewer generations of infection. This might be due to the short study period since the data for analysis were available for only 3 months. In France, data older than 3 months are deleted from the SORMAS database as demanded by the law. Some transmission chains may have continued to spread after the study period.

### Recommendations for Future Research

Further development of the SORMAS-Stats application can focus on the following features: (1) implement more epidemiological indicators that can inform the management of outbreaks such as statistics on hospitalization, immunization, or symptoms; (2) integrate data from other eHealth applications other than SORMAS; and (3) include outbreak detection, change point detection, or prediction models (eg, compartment models). In addition, as more public health workers use SORMAS-Stats, further research can investigate users’ experience, such as desirability, usefulness, and performance, through incorporating the concepts of human-computer interaction [31].

### Conclusions

We have provided an application for the real-time estimation of communicable disease parameters, which are essential for outbreak response. The use of the application requires only basic statistical analysis skills. SORMAS-Stats may greatly assist public health authorities in countries using SORMAS or similar tools by providing extensive visualizations, the computation of surveillance indicators, the estimation of epidemiological parameters, and the facilitation of the generation of routine epidemiological reports. This study also showcases how epidemiologists with skills in R statistical software programming only can build a web application, integrate it with a database of another application, and deploy it in the field for outbreak response.

## Abbreviations

k: dispersion parameter
SI: serial interval
SORMAS: Surveillance Outbreak Response Management and Analysis System
SORMAS-Stats: Surveillance Outbreak Response Management and Analysis System Statistical application
